# Letter to the Editor Re: McClure et al. Nutrients 2017, *9*, 95

**DOI:** 10.3390/nu9060585

**Published:** 2017-06-08

**Authors:** Suvi T. Itkonen, Christel J. E. Lamberg-Allardt

**Affiliations:** Calcium Research Unit, Division of Nutrition, Department of Food and Environmental Sciences, University of Helsinki, P.O. Box 66, 00014 Helsinki, Finland; christel.lamberg-allardt@helsinki.fi

**Keywords:** phosphorus, food additives, bioavailability, in vitro digestibility

Dear Editor, 

We read with interest the recently published paper by McClure et al. [1] that reports trends in intake and primary sources of dietary phosphorus in the NHANES data for the period 2001–2014. The authors raised a concern about grain products contributing an increasing proportion to total phosphorus intake in the typical American diet. Although providing intriguing data, the paper lacks an important element in evaluation of dietary phosphorus; the role of food additive phosphates and the bioavailability of phosphorus were not sufficiently addressed. 

Firstly, it is generally known that chemically analysed phosphorus contents of foodstuffs often differ from the values of food composition databases, especially for foods containing additive phosphates [2,3,4,5,6,7]. This causes inaccuracy and underestimation of phosphorus intakes. Secondly, the bioavailability of phosphorus differs among foodstuffs. Food additive phosphates have been shown to cause more adverse effects in calcium and bone metabolism in controlled studies than foods without added phosphates (e.g., [8,9,10]), indicating better bioavailability of food additive phosphates. Further, the phosphorus bioavailability from foodstuffs of animal origin has been demonstrated to be better than in vegetable-origin foodstuffs, where phosphorus mainly exists in the form of phytate (inositol hexakisphosphate), which needs to be hydrolysed to lower inositol phosphates to release the phosphorus in an inorganic, absorbable form [11]. 

Our research group developed an in vitro model that assesses the contents of digestible phosphorus (DP) that could reflect the bioavailability [12]. The model mimics the circumstances of the alimentary tract and the digestion process [12,13,14]. Our study on differently processed cereals showed that the in vitro DP content in bakery white wheat breads was 51% of total phosphorus, while in dark rye breads the corresponding proportion was 69–78% [12]. In the study of Karp et al. [13], DP contents among different plant-based foodstuffs differed substantially. Despite high total phosphorus content, legumes and seeds had low DP content compared with total phosphorus (6–42%), thus being relatively poor sources of bioavailable phosphorus. Among sweet bakery products, sweet buns (leavened with yeast) and cookies (containing no phosphate additives) had a total phosphorus content similar to that in white wheat bread. However, industrially prepared muffins containing phosphate additives (baking powder) differed from other wheat products; the proportion of DP was almost 100%. McClure et al. [1] have also noted this, but unfortunately, have not paid attention to the bioavailability of phosphorus from other foods. Considering other phosphate additive-containing foodstuffs, in cola beverages practically all phosphorus was digestible, however, the total phosphorus contents were only 10–16 mg/100 g, which is quite low [13]. Among animal-derived foods, processed cheeses contained 89–100% DP compared with total phosphorus, and overall, phosphorus additive-containing meat products had a higher proportion of DP than unenhanced products (up to 100% vs. 70–83%) [14]. Thus, the differing bioavailability of phosphorus should be taken into account and special attention should be paid to products that contain food additive phosphates when estimating the dietary phosphorus burden. Contrary to the argument of McClure et al. [1], in light of current knowledge, cereals/grains do not seem to be a particularly important source of bioavailable (absorbable) phosphorus, as also stated by Calvo and Uribarri [11]. 

Although cereals were the main source of phosphorus in the NHANES data [1], instead of highlighting cereals/grains as a risk product for excessive phosphorus intake, we suggest that the focus be on all food additive phosphorus sources, not only on bakery products. McClure and et al. [1] also refer to the paper of St-Jules and coworkers [15] that reviewed urinary phosphorus excretion in four human intervention studies that were not, however, designed to compare phosphorus bioavailability by food source. Despite the fact that St-Jules et al. found some discrepancies in the digestibility of food additive phosphates in those studies, they state that additives are the major dietary phosphorus source that can be eliminated without impairing diet quality of kidney patients [15]. With the growing evidence about the adverse health effects of dietary phosphorus not only in kidney patients but also in the general population [16,17], consumers’ consciousness about phosphorus must be raised. We agree with the authors that more controlled balance studies are required to better understand the bioavailability of phosphorus. There is also a need to further develop and standardize methods to analyze bioavailable phosphorus content in foodstuffs. 
